# Grievous Temporal and Occipital Injury Caused by a Bear Attack

**DOI:** 10.1155/2013/957251

**Published:** 2013-12-15

**Authors:** Sampath Chandra Prasad, Nikhil Dinaker Thada, Pallavi Rao, Smitha Rani Thada, Kishore Chandra Prasad

**Affiliations:** ^1^Department of Otolaryngology, Head & Neck Surgery, Kasturba Medical College, Manipal University, First Floor, Nethravathi Building, Balmatta, Mangalore, Karnataka 575001, India; ^2^Department of Radiodiagnosis, Kasturba Medical College, Manipal University, Attavar, Mangalore, Karnataka 575002, India; ^3^Department of Oral Medicine & Radiology, Manipal College of Dental Sciences, Manipal University, Madhava Nagar, Manipal, Karnataka 576104, India

## Abstract

Bear attacks are reported from nearly every part of the world. The chance of a human encountering a bear increases as the remote bear territory diminishes. The sloth bear is one of the three species of bears found in India, which inhabits the forests of India and its neighboring countries. Here we describe a teenager who came to us with a critical injury involving the face, temporal and occipital bones inflicted by a sloth bear attack. He underwent a temporal exploration, facial nerve decompression, pinna reconstruction, and occipital bone repair to save him from fatality.

## 1. Introduction

Bear attacks on humans are rare and are even more rarely reported in medical literature. Each year people have numerous accidental interactions with bears. A very small fraction of this results in human injury. The chance of a human encountering a bear increases as the remote bear territory diminishes. A search of scientific literature reveals very few articles detailing case reports or an in-depth analysis of injuries due to bear mauling. Here we discuss the presentation and subsequent management of an 18-year-old young man who came to us in a state of shock with a history of an assault by a sloth bear which led to an avulsion of his temporal and occipital bones associated with a facial nerve paralysis.

## 2. Case Report

An 18-year old teenager, a shepherd by occupation, presented to the emergency room in a state of shock with laceration of the ear and head, bleeding from the ear and the back of the head. A history by the relatives of the boy revealed that he had been mauled by a sloth bear in the forests of his native village where he had gone shepherding with his brother-in-law. To elaborate the account, which in itself is interesting, the two boys unexpectedly ran into a sloth bear in the forest, which attacked the brother-in-law. Seeing this, our boy ran to his rescue and in turn incurred the wrath of the bear on himself. While being mauled himself, the boy showed great presence of mind when, just as the bear was about to take a bite at his face, he hurled a rock piece from nearby into the bear's mouth and kicked it in its belly. The bear was frightened and ran way. The boy started bleeding profusely from his right ear and the back of his head and following that he collapsed into unconsciousness. He was taken to the village health center where he was given first aid but no fluid replacement or wound inspection was done. The boy was referred to our department after a 3-hour journey.

**Figure 1:**
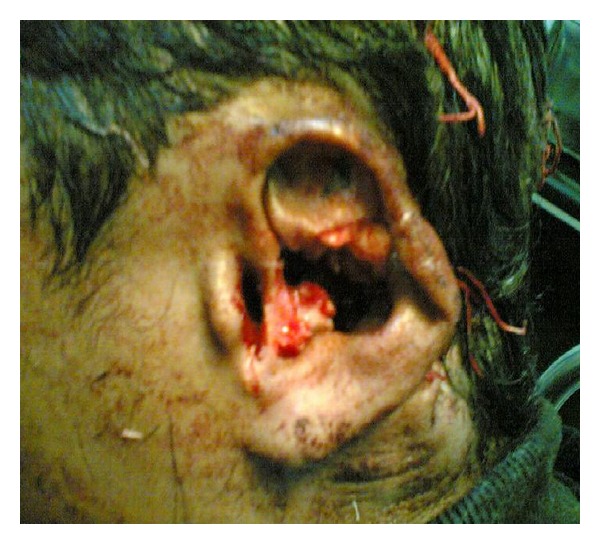
HB Grade IV facial nerve palsy.

**Figure 2:**
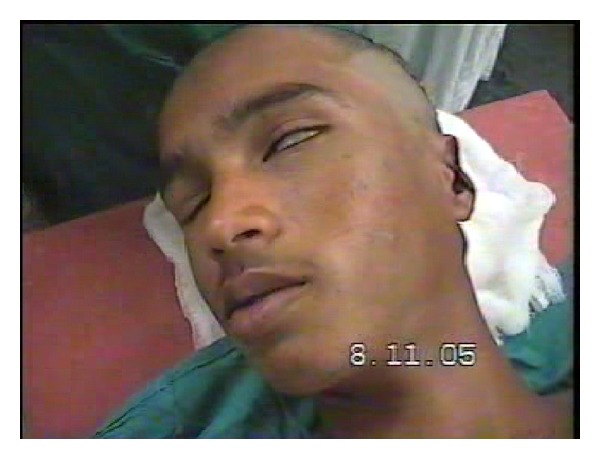
Pinna laceration.

There was no history of vomiting or nasal bleed. On examination, we found the patient to be semiconscious, disoriented, and in a state of hypovolemic shock. His Glasgow Coma Scale was 10/15. BP was 80/40; pulse was feeble with tachycardia, tachypnea, and cool clammy skin. There was active bleeding from the left ear wound and the scalp over the occipital bone. The patient had a painful spasm of the neck and was had House Brackmann (HB) Grade IV facial paralysis (Figure 1). A deep wide wound was identified in the left postaural region extending into the nape of the neck and the head. The left pinna was lacerated and mutilated (Figure 2). A large laceration was seen on the left parietal scalp. The external auditory canal (EAC), mastoid, squama of temporal bone, zygoma, and sternocleidomastoid (SCM) were exposed. The EAC skin was lacerated. The left mastoid bone and zygoma had multiple fractures. The TM showed bluish hue due to the presence of middle ear hematoma. Further inspection showed soft tissue injury to the parotid and the contents of the infratemporal fossa. Another laceration was identified over the occipital scalp on the left side through which the occipital bone was protruding out. On palpation, the occipital bone had multiple fractures. There was severe tenderness over the cervical spine and spasm of the paraspinal musculature. Rest of the physical examination was normal.

The patient was immediately put on intravenous fluid resuscitation with Ringer Lactate and Normal Saline and broad-spectrum antibiotics. An anti-rabies and tetanus vaccination was started. While a neurosurgery, orthopedic, and maxillofacial consultations were sought, the wounds were thoroughly debrided and bleeding points were identified and ligated leading to arrest of hemorrhage. The parietal scalp wound was primarily sutured under local anaesthesia. The neurosurgery team ruled out an intracranial lesion, the orthopedic team diagnosed the neck spasm and rigidity to be due to a severe spasm of the paraspinal musculature, and the maxillofacial team ruled out other facial fractures. The patient was advised critical observation for 24 hours and muscle relaxants. Three pints of blood was transfused subsequently.

**Figure 3:**
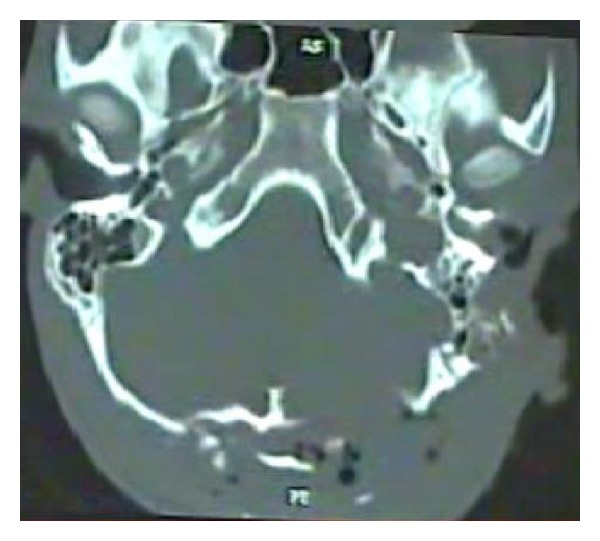
CT scan showing fracture of the left mastoid bone and comminuted fracture of the left occipital bone.

**Figure 4:**
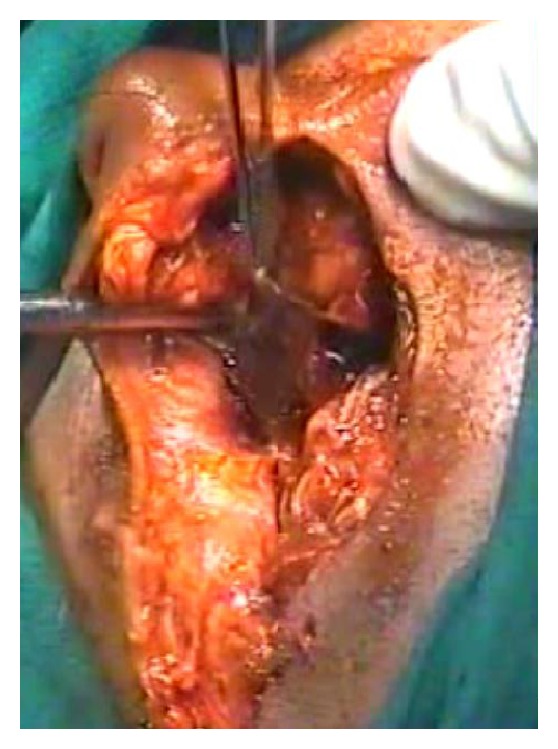
Deep wide wound in the postaural region with foliage recovered during debridement.

**Figure 5:**
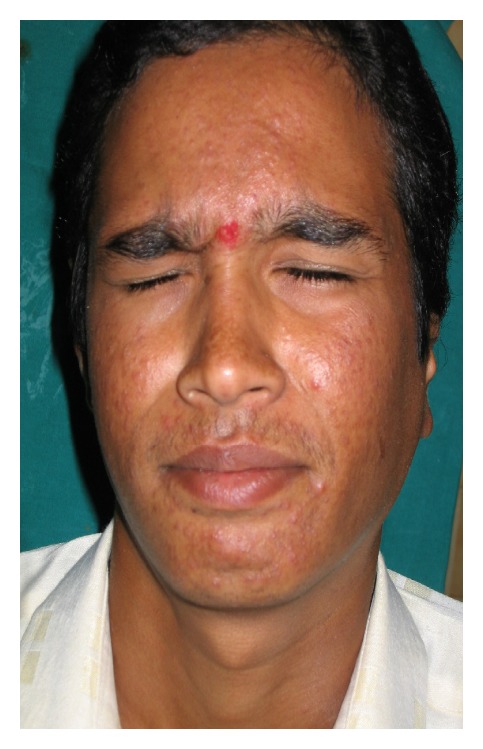
Complete recovery of facial palsy at 3 month follow-up.

**Figure 6:**
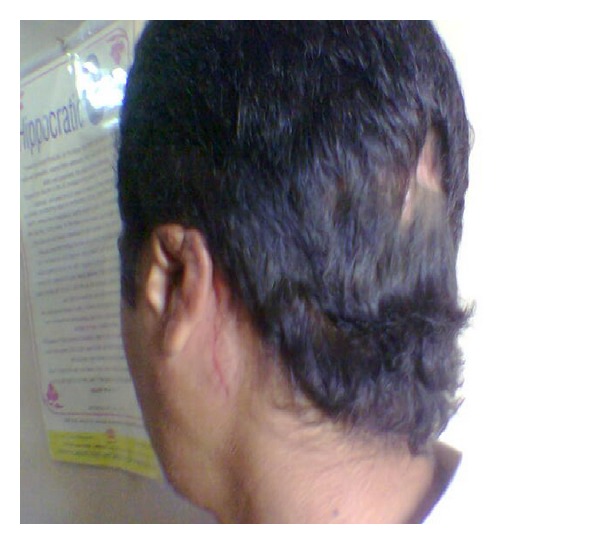
Well-healed occipital, parietal, and postaural wounds with residual disfigurement of the pinna.

A CT scan (Figure 3) done showed multiple fractures of the mastoid bone, a fracture of the zygoma, comminuted fracture of the occipital bone with no damage to the underlying dura or cerebral structures, compression of the facial nerve in its vertical part due to fractured bone in the fallopian canal, edema of the SCM, EAC, and parotid. There was no evidence of intracranial damage. The patient stabilized after 24 hrs following admission. After neurosurgical fitness was obtained, patient was immediately taken up for surgical exploration and wound debridement under GA. The temporal wound was thoroughly debrided. Material was sent for culture and sensitivity. Foliage and natural seeds were found impacted in the mutilated tissues (Figure 4). Tissue bits were sent for culture and sensitivity (C/S). The zygomatic fracture was reduced first. There were two fracture lines in the mastoid bone. An exploratory mastoidectomy was done followed by facial nerve decompression of the entire vertical part of the nerve. One of the fracture lines passed through this portion of the fallopian canal leading to compression of the nerve. Hematoma in the middle ear was suctioned out. The ossicles and the TM were found to be intact. Bits of lacerated SCM, parotid, and other tissues were excised. The halves of the lacerated pinna were sutured back. Skin edges were freshened and sutured together in layers. The occipital wound was then explored and thoroughly debrided. The occipital bone showed comminuted fractured with displaced bone chips. All the chips of bone were removed. The dura was inspected and found to be intact. The occipitalis muscle was rotated locally to form a flap to cover the defect. The skin was closed in two layers. The C/S isolated *Pseudomonas* sensitive to most anti-*Pseudomonas* drugs. The patient recovered remarkably over the next week. His facial nerve recovered to HB Grade II paralysis. He was discharged after 10 days. Follow-up after three months showed complete recovery of facial paralysis HB Grade I (Figure 5), with residual disfigurement of the pinna (Figure 6) and complete healing of all wounds.

## 3. Discussion

Though bear attacks are rare and incidents are often publicized in the media, even if few of the victims suffer serious injuries [1]. Not every otolaryngologist will come across a case of facial trauma due to bear mauling during a lifetime of practice. Though bear attacks are sporadically reported in India and around the world, an increasing frequency of such attacks has been noted by the residents in the forests of the Belthangady-Chickmagalur belt near Mangalore. We have, in fact, seen and treated four such cases, albeit less dramatic, in the last two years, where bear attacks were practically unheard of before. This is a clear indication of the now developing environmental conflict between humans and the animals in the wild due to loss of habitat for the latter. The degradation and loss of forests, especially outside protected areas, poses a major threat to sloth bear populations. Fragmentation of forests may lead to isolated, nonviable bear population [2]. Degradation, in the form of overgrazing, tree felling, fire, conversion, and reclamation for other uses, and over-extraction of forest resources that are essential for sloth bear survival appear to be occurring throughout the sloth bear range, particularly in the dry forests [3].

There are eight types of bears in the world. They include the American Black Bear, Brown Bear, Polar Bear, Giant Pandas, Asiatic Black Bear, sloth Bear, Spectacled Bear, and the Sun Bear. The sloth bears inhabit forests and tall grasslands in India, Nepal, Sri Lanka, and Bhutan. For those who frequent forests in India, sloth bear present a considerable danger—worse than that of tigers or leopards [3].

Bargali et al. [4] did a study in a forest division in India to describe sloth bear attacks and human injuries while defining an “attack” as an encounter that ends with human injury or death. His study observes that attacks were predominantly by a single bear (93%) and rarely by 2 (4%) or 3 bears. Most victims suffered multiple injuries (52%): single injuries on legs (25%), hand (12%), and head (8%). Also most of the bear attacks were in the monsoon season.

It is necessary to draw a protocol for the treating physician in the emergency room to act accordingly in case of an animal attack. This should be based on the following considerations. 

*(1) Identification*. Identify the animal (the subspecies) and determine if it is a domestic or wild animal. Several medical conditions place a patient at high risk of wound and rabies virus infection from a dog bite. Information that can help determine the patient's risk of infection includes the time of the injury, whether the animal was provoked, whereabouts of the animal (i.e., is it observable in quarantine?) and the general health, immunization status, and current location of the animal. In some locations, notification of animal control or local law enforcement may be necessary. Also, the patient's tetanus immunization status, current medications, and allergies must be noted in the record [5].

*(2) The Risk of Rabies*. The foremost danger that any wild animal poses to humans apart from physical insult is the danger of transmission of rabies. Postexposure prophylaxis (PEP) must be started immediately. This includes three components: wound care, passive immunization (immune globulin), and active immunization (vaccine). Both domestic and wild animals can transmit rabies. At one time, domestic animals, such as cats and dogs, were the main source of rabies bites. That situation has changed. Most cities, towns, and municipals now require that dogs be vaccinated for rabies. The number of dogs infected with the virus has dropped dramatically. Today, the vast majority of rabies cases in humans are caused by bites from wild animals, such as bats, raccoons, skunks, foxes, wolves, and coyotes. On 1st November, 1989, the first confirmed case of rabies in a polar bear (*Ursus maritimus*) was encountered by Inuit hunters in the vicinity of Cape Kendall, Southampton Island, Northwest Territories (Canada). The paper concluded that the impact of rabies on the population dynamics of polar bears probably is minimal and that rabies in polar bears constitutes a potential health hazard for polar bear hunters [6]. Further literature review suggests very little evidence to prove that bears could be carriers of the rabies virus though the possibility cannot be completely ruled out. Hence PEP is a must in all patients bit by wild animals.

*(3) Physical Injury*. The physical nature of injuries by animals can extend from trivial scratches to savage and mortal wounds. No site in the human body is protected from an animal assault. Different animals attack in different ways and this can be useful in determining injuries. Dog and cat bites can inflict cuts and lacerations, abrasions, crushing wounds, punctures, and fractured bones most commonly in the hands, wrists, and lower limbs. The central target area for the face includes the lips, nose, and cheeks. Bargali et al. [4] quoted that 8% of bear attacks were fatal. Most victims were attacked on their legs, 12.4% on their hands, and 11% on other parts of their bodies. 52% of cases of multiple injuries were reported. Victims suffered injuries such as fractures and severed body parts (eyes, scrotal sac). Big cats may cause superficial head and neck injuries, penetrating injuries to the neck and pharynx as well as a cervical spine fractures [7]. Injuries caused by large animals, such as a horse or a cow, require hospitalization and should be considered as high energy injuries [8]. The hallmark of a boar attack is the infliction of multiple penetrating injuries to the lower part of the body [9]. In a study of mammalian injuries, the hands and wrists are the most commonly involved sites as 36.1%, followed by the face, head and neck (22.6%), and the arms (13.7%) [10].

*(4) Bacteriology*. Wild animals carry a large number of bacteria in their mouth, and they may transmit a number of zoonotic diseases including viruses. Hence it is imperative that a microbiological analysis from wounds be done for all patients. Bears may carry rabies, hepatitis, distemper, *trichinella,* and other organisms. In one reported case, cultures of bacteria from a deep wound in the thigh grew *Streptococcus sanguis*, *Neisseria sicca, Bacillus *spp. and *Mycobacterium fortuitum* [11] *Serratia fonticola*, *Serratia marcescens*, *Aeromonas hydrophila*, *Bacillus cereus*, and *Enterococcus durans* have also been isolated from bear wounds [12]. The large cats, such as leopards and cougars, may carry the *Pasteurella* organism and they are very difficult to tame. Cats and dogs are reported to carry such diseases as Bartonella, Tularemia, and *Pasturella multocida* organisms as well as cat scratch fever.

*(5) Management*. Most wounds can be treated in the ER. Essentials of treatment are necessary inspection, debridement, irrigation, and closure, if indicated. Wounds are inspected to identify deep injury and devitalized tissue preferably under GA. Care should be taken to visualize the bottom of the wound. Debridement is an effective means of preventing infection. Removing devitalized tissue, particulate matter, and clots prevents these from becoming a source of infection, much like any foreign body. Clean surgical wound edges result in smaller scars and promote faster healing. Irrigation with saline or antibiotic solution is another important means of infection prevention. Heavily contaminated wounds require more irrigation. Primary closure is considered in relatively clean bite wounds or wounds that can be cleansed effectively. Others are best treated by delayed primary closure. Head and neck wounds have a very low infection risk [13, 14]. Bite wounds to the lower extremities, with a delay in presentation, or in immunocompromised hosts generally should be left open. Tetanus and rabies prophylaxis should be given for all wounds from wild animals. Antibiotics should always be a broad-spectrum one (authors use Amoxicillin/clavulanate with 3rd generation cephalosporins) with subsequent alterations depending on the C/S report. Treatment of animal bites involves multiple organs and appropriate consultations must be given for further specialized management. Facial nerve injury is a rare occurrence unless the attack involves the temporal bone like our case. Facial nerve decompression should be done wasting as little time as possible, preferably in the first 24 hrs to get the best results. Many animal bite wounds result in disfiguring scars, which require reconstructive plastic surgery.

*(6) Follow-Up*. Patients should be given detailed instructions on signs of infection and rabies and advised to return to the department in such a case.

*(7) Psychological Assessment*. A victim may also suffer psychological problems as a result of an attack, such as developing a fear of animals or being afraid to go out alone. The psychological impact of a vicious animal bite can be severe and long lasting and may require treatment by a psychologist or psychiatrist.

Travelers, tourists, and workers in unfamiliar areas should all be aware of the potential for bites and their consequences. Peace Corps volunteers, missionaries, and others should be prepared for such an occurrence and have appropriate instruction and preventive measures considered prior to their travel. During these considerations, they should also review the various diseases that may be transmitted through these bites. Warm-blooded animal bites occur worldwide from a large number and variety of animals. Each animal species may carry its own particular peril and injury potential. Treatment depends on the type of animal and the bite. Awareness and prevention are primary in the avoidance of major injury and healthcare risks [14].

## 4. Conclusion

While it is true that bears have the potential to be dangerous to humans and that a number of people are injured by bears every year, in reality, the incidence of attacks, on humans is relatively rare. Wild-animal attacks, though rare, remind us that humans can still be food or prey. Characteristic patterns of injury and wound infection should be appropriately identified and treated. Though bear bites causing rabies is not definitively reported and researched, it is important to realize that all mammals have the potential to carry and transmit rabies and PEP must be started immediately. Primary closure of wounds gives good results, especially in the head and neck. The conflict of human and animal environments due to man's excessive needs and greed will lead to more situations like this and it is necessary that the treating doctor be aware of such conditions and the treatment protocols to provide optimum care in such cases. Awareness, education, knowledge, and prevention, rather than the elimination of animal populations, may be the best way to control wild-animal attacks on humans in the future.
